# Selected hematologic and biochemical measurements in African HIV-infected and uninfected pregnant women and their infants: the HIV Prevention Trials Network 024 protocol

**DOI:** 10.1186/1471-2431-9-49

**Published:** 2009-08-07

**Authors:** Kasonde Mwinga, Sten H Vermund, Ying Q Chen, Anthony Mwatha, Jennifer S Read, Willy Urassa, Nicole Carpenetti, Megan Valentine, Robert L Goldenberg

**Affiliations:** 1 Department of Paediatrics of the University Teaching Hospital and the University of Zambia School of Medicine, and the Centre for Infectious Disease Research in Zambia, Lusaka, Zambia; 2 Now with the World Health Organization, Lusaka; 3 Institute for Global Health and Department of Pediatrics, Vanderbilt University School of Medicine, Nashville, TN, USA; 4 Statistical Center for HIV/AIDS Research and Prevention, Fred Hutchinson Cancer Research Center, Seattle, WA, USA; 5 National Institute of Child Health and Human Development, National Institutes of Health, Bethesda, MD; 6 Muhimbili University, Dar-es-Salaam, Tanzania; 7 College of Medicine – Johns Hopkins University Research Project, Blantyre, Malawi; 8 Family Health International, Chapel Hill, NC, USA; 9 Department of Obstetrics and Gynecology, Drexel University College of Medicine, Philadelphia, PA, USA

## Abstract

**Background:**

Reference values for hematological and biochemical assays in pregnant women and in newborn infants are based primarily on Caucasian populations. Normative data are limited for populations in sub-Saharan Africa, especially comparing women with and without HIV infection, and comparing infants with and without HIV infection or HIV exposure.

**Methods:**

We determined HIV status and selected hematological and biochemical measurements in women at 20–24 weeks and at 36 weeks gestation, and in infants at birth and 4–6 weeks of age. All were recruited within a randomized clinical trial of antibiotics to prevent chorioamnionitis-associated mother-to-child transmission of HIV (HPTN024). We report nearly complete laboratory data on 2,292 HIV-infected and 367 HIV-uninfected pregnant African women who were representative of the public clinics from which the women were recruited. Nearly all the HIV-infected mothers received nevirapine prophylaxis at the time of labor, as did their infants after birth (always within 72 hours of birth, but typically within just a few hours at the four study sites in Malawi (2 sites), Tanzania, and Zambia.

**Results:**

HIV-infected pregnant women had lower red blood cell counts, hemoglobin, hematocrit, and white blood cell counts than HIV-uninfected women. Platelet and monocyte counts were higher among HIV-infected women at both time points. At the 4–6-week visit, HIV-infected infants had lower hemoglobin, hematocrit and white blood cell counts than uninfected infants. Platelet counts were lower in HIV-infected infants than HIV-uninfected infants, both at birth and at 4–6 weeks of age. At 4–6 weeks, HIV-infected infants had higher alanine aminotransferase measures than uninfected infants.

**Conclusion:**

Normative data in pregnant African women and their newborn infants are needed to guide the large-scale HIV care and treatment programs being scaled up throughout the continent. These laboratory measures will help interpret clinical data and assist in patient monitoring in a sub-Saharan Africa context.

**Trial Registration:**

nicalTrials.gov Identifier NCT00021671.

## Introduction

Hematological parameters are affected by many factors, including age, sex, diet, recent nutritional status, and consumption of medications or illicit drugs.[1-4] Reference values for hematological and biochemical assays in pregnant women and in infants are based largely on data from Caucasian populations. Normative data have been reported from only a few populations living in sub-Saharan Africa.[2,4-10] Fewer studies still highlight expected values from women and infants with HIV infection or exposure.[3,11-13] Within a large clinical trial of the prevention of mother-to-child transmission of HIV, we took advantage of the fact that we had laboratories that were certified by the National Institutes of Health performing laboratory assessments with a high degree of oversight and quality control. All laboratories were subject to rigorous monitoring, including receipt of proficiency panels. Thus, we were in an excellent position to provide laboratory data from both pregnant women and their newborn offspring within a high quality laboratory environment in four African cities. These data were collected to assess the safety of the antibiotic intervention of the parent clinical trial and the status of the patient vis-à-vis HIV infection or risk. We describe hematological and biochemical measures and trends over time in a large cohort of pregnant African women and infants with and without human immunodeficiency virus type 1 (HIV) infection. Given the magnitude of the current efforts to identify and treat HIV disease in Africa, we believe that data from our large sample will prove helpful to service providers seeking to interpret data from their own pregnant clinical subjects and their infants.

## Patients and Methods

### The HIV Prevention Trials Network Protocol 024 Trial

The HIV Prevention Trials Network Protocol 024 (HPTN024) study was a Phase III randomized, double blind, placebo-controlled clinical trial of antibiotics to reduce chorioamnionitis-associated mother-to-child transmission of HIV. The trial was conducted in four African sites: Blantyre, Malawi, Lilongwe, Malawi; Dar-es-Salaam, Tanzania; and Lusaka, Zambia. Prior to initiation of the trial, approval was received from institutional review boards or ethics committees at all participating sites and universities. The findings of the HPTN024 trial itself have been published showing that an antepartum and peripartum antibiotic regimen did not reduce the risk of MTCT of HIV-1 in African women with high prevalence rates of bacterial vaginosis and subclinical chorioamnionitis.[14] HIV-infected and HIV-uninfected women were enrolled at 20–24 weeks gestation from antenatal clinics from July 2001 to February 2003. By trial design, in three of the four sites, there were five pregnant HIV-infected women enrolled in the study for each pregnant HIV-uninfected woman. The principal exclusion criteria were related to serious illness that would prevent the woman from participating in a research study. Otherwise, subjects were highly representative of the public sector clinics from which they were recruited. All laboratory data indicated for the parent protocol were obtained on each subject, except that viral loads were not obtained if the subject was HIV-uninfected.

Enrolled women were randomized to receive either metronidazole 250 mg and erythromycin 250 mg every 8 hours for 7 days at 20–24 weeks, followed by metronidazole 250 mg and ampicillin 500 mg every 4 hours with onset of premature rupture of membranes or labor, or identically appearing placebos. Follow-up study visits occurred at 28 weeks and 36 weeks gestation. Infant study visits were conducted at birth and at 4–6 weeks of age. All women received a conventional iron-containing vitamin/mineral preparation designed for pregnant women (Tishcon Corporation, Baltimore, MD) daily from enrolment and until delivery. The supplement included: 30 mg iron, 400 mcg folic acid, 5000 IU vitamin A, 400 IU vitamin D, 30 IU vitamin E, 50 mg vitamin C, 2 mg vitamin B1, 3 mg vitamin B2, 3 mg vitamin B6, 5 mcg vitamin B12, 20 mg niacin, 250 mg calcium, 150 mcg iodine, 100 mg magnesium, and 15 mg zinc.

HIV-infected women were offered single dose nevirapine (NVP) prophylaxis at delivery for prevention of mother-to-child transmission of HIV. Infants of HIV-infected women received NVP within 72 hours of birth. No other antiretroviral drugs were used by mothers and infants in the trial, as these drugs were unavailable in the study settings at the time.

All seroconverters had their baseline stored specimens sampled by PCR so that persons who were acutely infected at baseline could be excluded. Hence, we are certain that our seronegative women were not acutely infected. Furthermore, these baseline data were collected within a month of enrolment, and often on the same day as enrolment.

Since the clinicians were blinded to the randomized treatment assignment, women were treated for all infections per the local treatment guidelines. The receipt of non-study antibiotics and all other medications received by the women was recorded using open-ended questions in the concomitant medications log form (data not presented).

We used structured questionnaires to collect information on maternal demographics as well as medical, obstetric and sexual histories. We estimated infant gestational age through the neuromuscular and physical maturity indices of the new Ballard examination. Trained nurses assessed birth weights.

For infants born to the study women, only the first live born infant was included in this analysis. If twins were born, only the firstborn twin was included (since laboratory outcomes for twins are likely to be correlated). Women could not be enrolled for subsequent pregnancies if they had already enrolled previously.

### Laboratory Procedures

Venous blood was collected from pregnant women at both the 20- to 24-week visit and the 36-week visit. Blood for complete blood counts (CBCs) was collected in ethylenediaminetetraacetic acid (EDTA) vacutainers and analyzed at local laboratories on Coulter machines. Blood for alanine aminotransferase (ALT) assays was stored in EDTA vacutainers. Infant blood was collected by heel stick. CBCs and lymphocyte subsets (counts and percentages) were determined at both visits using HPTN Central Lab-approved site-specific procedures. Analyses for CD4+ and CD8+ T lymphocyte cell counts were done using FACSCount™ flow cytometer in three sites (Dar-es-Salaam, Lilongwe and Lusaka) while one site (Blantyre) used FACScan™ flow cytometer. All analyses were done according to the manufacturer's procedures. For infants, CBCs were obtained at both the birth visit and the 4–6-week visit. At the 4–6-week visit, ALT assays were performed with chemistry analyzers according to manufacturer's protocols.

Blood samples from the pregnant women were screened for HIV at local laboratories using two different enzyme linked immunosorbent assays (ELISA), and confirmed with Western blot assays. The HIV infection status of infants at birth and 4–6 weeks of age was determined by analyzing dried blood spots, using a polymerase chain reaction (PCR)-based HIV RNA assay. Results from ELISA assays (for women) and HIV RNA assays (for infants) were confirmed centrally.

Infants were categorized as HIV-uninfected at birth if they had a negative HIV RNA assay at birth, or if they were missing an HIV RNA result from the birth visit, but had a negative HIV RNA test at 4–6 weeks of age. Infants who had a negative HIV RNA test at birth, but a positive HIV RNA test at 4–6 weeks were categorized as HIV-uninfected at birth, and as HIV-infected at 4–6 weeks of age.

The HPTN Central Laboratory at Johns Hopkins University reviewed and certified all local laboratories before the initiation of the trial. The Central Laboratory verified virological, serological, hematological, immunological, and biochemical tests based on proficiency panels provided by the College of American Pathology (CAP) and United Kingdom (U.K.) National External Quality Assessment Service (UKNEQAS) on a periodic basis throughout the trial.

### Statistical Analysis

We summarized all laboratory values by their means, 95% confidence intervals (2.5^th ^and 97.5^th ^percentiles), and calculated their associated standard deviations for HIV-infected and HIV-uninfected women and infants separately. Two-sample comparisons were done by hypothesis testing on continuous and categorical outcomes using the Student's t-test and the Chi-square test (or the Fisher's exact test, when indicated), respectively. Comparison on changes in laboratory measures between visits (20–24 weeks and 36 weeks) was done by hypothesis testing using paired sample t-tests. Similar analyses were also performed for infants (birth visit and 4–6 week visit).

## Results

### Size and Characteristics of the Study Population

**Figure 1:**
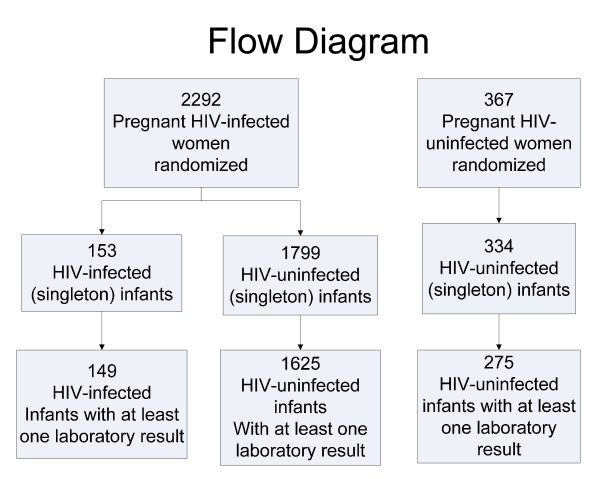
**Patient recruitment and retention for the HIV Prevention Trials Network 024 randomized clinical trial protocol in Malawi, Tanzania, and Zambia**.

We enrolled 2,659 eligible pregnant women into HPTN024. Of these 2,659 women, 2,292 (86%) were HIV-infected and 367 (14%) were uninfected (Figure 1). There were 2,382 live born infants (2,052 born to HIV-infected women and 331 born to uninfected women). The mean gestational age at birth (n = 2382) was 38.4 (± 0.06) weeks using the new Ballard score. The mean birth weight in the study was 2,957 (± 11.8) grams. Infants born to HIV-infected mothers had a 37-gram lower mean birth weight compared to infants born to HIV-uninfected mothers (2980 grams ± 505; 95% ci: 2957–3005 vs. 3079 grams ± 515; 95% ci: 3023–3135; respectively; p = 0.002 by Student's t test). The mean head circumference was 34.6 (± 0.05) cm. The mean Apgar score at 1 minute was 8.2 (± 0.03) and was 9.6 (± 0.03) at 5 minutes.

Of the 2,052 infants born to HIV-infected women, receipt of NVP prophylaxis was as follows: both mother and infant received NVP (1,817), mother received NVP but infant did not (143), mother did not receive NVP but infant did (52), and neither mother nor infant received NVP (40 pairs). None of the infants born to HIV-uninfected mothers was exposed to NVP.

HIV-infected women were, on average, two years older than HIV-uninfected women (p < 0.001); education levels were comparable (Table 1). Infants of HIV-infected women had lower birth weights than those born to HIV-uninfected women: 2,936 (± 12.9) grams vs. 3,090 (± 28.2) grams, respectively (p < 0.0001). Mean head circumference was also lower among infants of HIV-infected women compared to the mean head circumferences of those born to HIV-uninfected women (Table 1).

**Table 1:** Selected characteristics of pregnant women and their infants according to maternal HIV infection status

**Pregnant women in antenatal care**	**HIV-infected women**	**HIV-uninfected women**	**P value**
		
	N = 2292	n = 367	
Mean age in years (± SD)	25.2 (0.1)	23.2 (0.3)	< 0.0001
Able to read %	77.3%	79.8%	0.3
Homemakers %	77.7%	86.6%	0.0002
Mean years of education (± SD)	6.6 (0.1)	6.8 (0.2)	0.2
Married or living with partner %	91.5%	96.7%	< 0.0005
Spouse with formal employment %	54.6%	56.6%	0.8
Spouse's years of education (± SD)	9.2 (0.1)	9.3 (0.2)	0.5
Lives in own house %	25.7%	31.3%	0.0015
Electricity in the house %	40.5%	33.8%	0.02
Running water %	42.1%	33.8%	0.003

Infants born to enrolled women*	**Mother HIV-infected**	**Mother HIV-uninfected**	**P value**
		
	N = 2052	N = 331	

Mean birth weight in grams (± SD)	2936.1 (12.9)	3090.4 (28.2)	< 0.0001
Mean Apgar score at 5 min (± SD)	9.6 (< 0.01)	9.6 (0.1)	0.4
Mean gestational age in weeks (± SD)	38.4 (0.1)	38.4 (0.1)	0.9
Mean head circumference (± SD)	34.5 (0.1)	34.9 (0.1)	0.02
Male sex %	50.5%	48.0%	0.4

* Live born infants; if twin, then first born only

### Laboratory Measures in Pregnant Women

Tables 2 and 3 compare the hematological values for HIV-infected versus HIV-uninfected pregnant women at 20–24 weeks (Table 2) and at 36 weeks (Table 3). At both 20–24 weeks and 36 weeks, HIV-infected pregnant women had lower red blood cell (RBC) counts, hemoglobin, hematocrit, and WBC counts than HIV-uninfected women. Platelet counts and the monocyte differential counts were higher in HIV-infected women than in HIV-uninfected women at both time points. The CD4+ T lymphocyte cell counts (CD4+ cell count) were lower in HIV-infected women compared to HIV-uninfected women. From the paired samples' t-test (comparing the changes in CD4+ cell count at 20–24 weeks vs. 36 weeks), the CD4+ cell counts increased during pregnancy among the HIV-infected women (p = 0.0002), while there was no significant change in this measure among the HIV-uninfected women. The mean of the paired differences of CD4+ cell counts among HIV-infected women was +14.8 cells/μL from the 20–24-week visit compared to the 36-week antenatal visit.

**Table 2:** Laboratory values among HIV-infected and HIV-uninfected pregnant women at 20–24 weeks gestation

**Laboratory Parameter**	**HIV-infected**				**HIV-uninfected**				p value*
	**n**	**Mean ± SD**	**95% CI**		**n**	**Mean ± SD**	**95% CI**		
			**Lower**	**Upper**			**Lower**	**Upper**	
Red blood cell count 10^6^/μL	2266	**3.6 ± 0.5**	2.56	4.65	367	**3.8 ± 0.6**	2.65	4.92	< 0.0001
Hemoglobin g/dl	2262	**10.1 ± 1.4**	7.1	12.7	367	**11.0 ± 1.3**	8.2	13.2	< 0.0001
Hematocrit %	2265	**29.9 ± 4.2**	21.4	37.7	367	**32.3 ± 4.6**	23.8	39.4	< 0.0001
Platelets 10^3^/μL	2267	**231.6 ± 79.9**	102	410	367	**204.3 ± 64.9**	97	350	< 0.0001
White blood cell count 10^3^/μL	2266	**6.1 ± 2.0**	2.9	10.3	366	**6.9 ± 1.9**	3.3	11.1	< 0.0001
Lymphocytes %	2197	**31.0 ± 9.9**	16.4	58.1	358	**31.5 ± 8.7**	19	57	0.4
Monocytes %	2197	**8.1 ± 5.0**	1.9	20.9	358	**6.8 ± 4.4**	1.9	17	< 0.0001
Granulocytes %	2198	**60.7 ± 12.5**	22.8	78.6	358	**61.7 ± 10.8**	28.6	77.5	0.1
CD4+ T lymphocyte count cells/μL	2072	**374.4 ± 214.6**	64	899	246	**809.6 ± 257.9**	344	1366	< 0.0001
CD8+ T lymphocyte count cells/μL	2072	**771.1 ± 357.6**	276	1674	246	**513.5 ± 196.8**	262	1002	< 0.0001
CD4+ T lymphocyte %	287	**23.3 ± 10.7**	5	46	55	**47.6 ± 8.4**	33	65	< 0.0001
CD8+ T lymphocyte %	287	**55.4 ± 15.3**	26	83	55	**31.7 ± 8.4**	18	50	< 0.0001
Total T lymphocytes as a % of all lymphocytes	235	**74.5 ± 14.4**	21	94	47	**75.6 ± 8.3**	60	93	0.6

*P values by Student's t test comparing laboratory values of HIV-infected and HIV-uninfected pregnant women at 20–24 weeks gestation.

**Table 3:** Laboratory values among HIV-infected and HIV-uninfected pregnant women at 36 weeks gestation

**Laboratory Parameter**	**HIV-infected**				**HIV-uninfected**				**p value**
	**n**	**Mean ± SD**	**95% CI**		**n**	**Mean ± SD**	**95% CI**		
			**Lower**	**Upper**			**Lower**	**Upper**	
Red blood cell count 10^6^/μL	1643	**3.8 ± 0.6**	2.78	4.94	280	**3.9 ± 0.7**	2.865	5.34	< 0.0001
Hemoglobin g/dl	1642	**10.7 ± 1.4**	7.9	13.2	280	**11.3 ± 1.3**	9	13.95	< 0.0001
Hematocrit %	1643	**31.7 ± 4.3**	23.3	39.5	280	**33.3 ± 4.2**	25.9	41.85	< 0.0001
Platelets 10^3^/μL	1642	**209.8 ± 79.1**	88	402	280	**182.9 ± 64.8**	86.5	344.5	< 0.0001
White blood cell count 10^3^/μL	1639	**6.1 ± 2.0**	3.1	10.2	280	**6.7 ± 2.1**	3.8	11.2	< 0.0001
Lymphocytes %	1633	**31.4 ± 8.7**	16.7	50.5	274	**31.8 ± 8.4**	18.1	53.6	0.5
Monocytes %	1633	**8.4 ± 5.1**	2.1	21.9	274	**7.5 ± 5.3**	1.1	23.1	0.01
Granulocytes %	1632	**60.1 ± 11.0**	32.1	77.8	274	**60.7 ± 10.8**	32.6	76.5	0.4
CD4+ T lymphocyte count cells/μμL	1459	**399.8 ± 222.7**	65	901	166	**786.0 ± 266.4**	368	1325	< 0.0001
CD8+ T lymphocyte count cells/μL	1459	**776.2 ± 336.3**	293	1618	166	**506.8 ± 218.5**	216	1106	< 0.0001
CD4+ T lymphocyte %	185	**24.2 ± 10.4**	4	43	49	**45.0 ± 9.3**	28	66	< 0.0001
CD8+ lymphocyte %	185	**54.0 ± 12.5**	32	79	49	**31.1 ± 7.4**	21	43	< 0.0001
Total T lymphocytes as a % of all lymphocytes	173	**77.5 ± 9.7**	57	92	43	**74.1 ± 9.6**	54	90	0.04

Note: Indicates t-test P values comparing laboratory values between HIV-infected and HIV-uninfected women at 36 weeks gestation

About one-quarter of the women who had been seen at the 20–24-week visit were not seen at the 36-week visit. The mean maternal age for women who were seen only at 20–24 weeks was 24.5 ± 5.0 years, while women seen both at gestational age 20–24 weeks as well as at 36 weeks were six months older, on average (mean age of 25.1 ± 4.8 years; p = 0.004). Log viral load of women who were seen only at 20–24 weeks was 4.39 ± 0.78 log_10 _copies/mL while women seen both at gestational age 20–24 weeks as well as at 36 weeks had a 0.15 log_10 _copies/mL lower mean viral load of 4.24 ± 0.82 log_10 _copies/mL (p = 0.0002). We highlight the relevance of this observation, related to the CD4+ cell count changes in the discussion.

We found the differences between HIV-infected and uninfected pregnant women to persist, for the most part, throughout the range of CD4+ cell counts (Tables 4, 5, 6 and 7). For example, even women with HIV infection and CD4+ cell counts >500/μL had lower hemoglobins and higher platelet counts than did HIV-uninfected women (Table 7). As one would expect, hematogical parameters were more aberrant in women with lowest CD4+ cell counts (Table 4).

**Table 4:** Comparison of HIV-infected women with CD4+ cell counts <200/μL to HIV-uninfected women

	**HIV (+) CD4 < 200/μL**				**HIV (-)**				
**Laboratory measures**	**Mean**	N	P2.75	P97.5	**Mean**	N	P2.75	P97.5	**P value**
	**Study visit at 20–24 weeks gestation**								
Red blood cells 10^6^/μL	**3.4**	448	2.4	4.6	**3.8**	367	2.9	4.9	**< .0001**
Hemoglobin g/dL	**9.6**	447	7.0	12.0	**11.0**	367	8.2	13.2	**< .0001**
Hematocrit %	**28.5**	447	21.3	35.9	**32.3**	367	23.8	39.4	**< .0001**
Platelets 10^3^/μL	**226.0**	449	100	391	**204.3**	367	97	350	**< .0001**
White blood cells 10^3^/μL	**5.2**	449	2.7	9	**6.9**	366	3.3	11.1	**< .0001**
Lymphocytes %	**28.0**	435	12.8	55.1	**31.5**	358	19	57	**< .0001**
Monocytes %	**8.9**	435	2.4	23.8	**6.8**	358	1.9	17	**< .0001**
Granulocytes %	**63.0**	436	24	81.8	**61.7**	358	28.6	77.5	**0.1**
CD4+ cells/μL	**130**	456	26	198	**809.6**	246	344	1366	**< .0001**
CD8+ cells/μL	**661**	456	192	1615	**513.5**	246	262	1002	**< .0001**
CD4 %	**14.0**	89	3	29	**47.6**	55	33	65	**< .0001**
CD8 %	**59.7**	89	15	88	**31.7**	55	18	50	**< .0001**
Total T-Lymphocytes as % of all lymphocytes	**69.6**	76	17	92	**75.6**	47	60	93	**0.04**

	**Study visit at 36 weeks gestation**								

Red blood cells 10^6^/μL	**3.7**	279	2.6	4.9	**3.9**	280	2.9	5.3	**< .0001**
Hemoglobin g/dL	**10.4**	279	7.4	13.2	**11.3**	280	9.0	14.0	**< .0001**
Hematocrit %	**30.8**	279	22.6	39.0	**33.3**	280	25.9	41.9	**< .0001**
Platelets 10^3^/μL	**208.8**	279	77	419	**182.9**	280	87	345	**< .0001**
White blood cells 10^3^/μL	**5.0**	279	2.4	8.5	**6.7**	280	3.8	11.2	**< .0001**
Lymphocytes %	**28.7**	279	13.5	51.6	**31.8**	274	18.1	53.6	**< .0001**
Monocytes %	**8.8**	279	2.6	21.9	**7.5**	274	1.1	23.1	**0.003**
Granulocytes %	**62.5**	279	35.6	80.4	**60.7**	274	32.6	76.5	**0.06**
CD4+ cells/μL	**131**	280	19	196	**786**	166	368	1325	**< .0001**
CD8+ cells/μL	**656**	280	215	1524	**507**	166	216	1106	**< .0001**
CD4 %	**13.8**	49	3	29	**45.0**	49	28	66	**< .0001**
CD8 %	**60.2**	49	34	88	**31.1**	49	21	43	**< .0001**
Total T-Lymphocytes as % of all lymphocytes	**74.9**	47	50	93	**74.1**	43	54	90	**0.7**

Laboratory values for pregnant women at the 20–24-week gestational age visit and at the 36-week visit. Of the 2633 women with data at baseline, 727 (27.6%) were missing week 36 data and 1906 (72.4%) had 36-week data. It is possible that week 36 women were slightly healthier than week 20–24 women, since the 36 week women had a slightly higher CD4+ cell count and may have been more likely to be able to keep the 36-week appointment (see discussion).

Lower 95% confidence interval = P2.75 = 2.75%ile

Upper 95% confidence interval = P97.5 = 97.5%ile

**Table 5:** Comparison of HIV-infected women with CD4+ cell counts 200–349/μL to HIV-uninfected women

	**HIV (+) CD4 200–349/μL**				**HIV (-)**				
**Laboratory measures**	**Mean**	N	P2.75	P97.5	**Mean**	N	P2.75	P97.5	**P value**
**Study visit at 20–24 weeks gestation**									
Red blood cells 10^6^/μL	**3.5**	608	2.53	4.52	**3.8**	367	2.65	4.92	**< .0001**
Hemoglobin g/dL	**10.0**	607	6.7	12.2	**11.0**	367	8.2	13.2	**< .0001**
Hematocrit %	**29.5**	608	21	36.4	**32.3**	367	23.8	39.4	**< .0001**
Platelets 10^3^/μL	**231.9**	608	101	413	**204.3**	367	97	350	**< .0001**
White blood cells 10^3^/μL	**5.7**	608	2.6	9.1	**6.9**	366	3.3	11.1	**< .0001**
Lymphocytes %	**30.4**	597	16.4	58.1	**31.5**	358	19	57	**0.0778**
Monocytes %	**8.4**	597	2.1	21.4	**6.8**	358	1.9	17	**< .0001**
Granulocytes %	**60.8**	597	21.1	78.7	**61.7**	358	28.6	77.5	**0.3**
CD4 cells/μL	**272.8**	612	202	346	**809.6**	246	344	1366	**< .0001**
CD8 cells/μL	**737.0**	612	284	1589	**513.5**	246	262	1002	**< .0001**
CD4 %	**22.7**	92	12	37	**47.6**	55	33	65	**< .0001**
CD8 %	**57.3**	92	31	82	**31.7**	55	18	50	**< .0001**
Total T-Lymphocytes as % of all lymphocytes	**76.4**	75	48	98	**75.6**	47	60	93	**0.7**

**Study visit at 36 weeks gestation**									

Red blood cells 10^6^/μL	**3.7**	391	2.75	4.87	**3.9**	280	2.865	5.34	**< .0001**
Hemoglobin g/dL	**10.5**	391	7.9	13	**11.3**	280	9	13.95	**< .0001**
Hematocrit %	**31.1**	391	22.4	38.5	**33.3**	280	25.9	41.85	**< .0001**
Platelets 10^3^/μL	**201.6**	391	88	356	**182.9**	280	86.5	344.5	**0.0005**
White blood cells 10^3^/μL	**5.6**	391	3.3	8.9	**6.7**	280	3.8	11.2	**< .0001**
Lymphocytes %	**30.8**	387	16.2	50.7	**31.8**	274	18.1	53.6	**0.1**
Monocytes %	**8.8**	387	2.4	22.5	**7.5**	274	1.1	23.1	**0.0020**
Granulocytes %	**60.2**	387	30.4	78.5	**60.7**	274	32.6	76.5	**0.6**
CD4+ cells/μL	**274.8**	395	202	346	**786.0**	166	368	1325	**< .0001**
CD8+ cells/μL	**757.2**	395	313	1731	**506.8**	166	216	1106	**< .0001**
CD4 %	**24.3**	66	13	42	**45.0**	49	28	66	**< .0001**
CD8 %	**54.1**	66	32	73	**31.1**	49	21	43	**< .0001**
Total T-Lymphocytes as % of all lymphocytes	**78.0**	63	61	91	**74.1**	43	54	90	**0.02**

Laboratory values for pregnant women at the 20–24-week gestational age visit and at the 36-week visit. Of the 2633 women with data at baseline, 727 (27.6%) were missing week 36 data and 1906 (72.4%) had 36-week data. It is possible that week 36 women were slightly healthier than week 20–24 women, since the 36 week women had a slightly higher CD4+ cell count and may have been more likely to be able to keep the 36-week appointment (see discussion).

Lower 95% confidence interval = P2.75 = 2.75%ile

Upper 95% confidence interval = P97.5 = 97.5%ile

**Table 6:** Comparison of HIV-infected women with CD4+ cell counts 350–500/μL to HIV-uninfected women

	**HIV (+) CD4 350–500/μL**				**HIV (-)**				
**Laboratory measures**	**Mean**	N	P2.75	P97.5	**Mean**	N	P2.75	P97.5	**P value**
**Study visit at 20–24 weeks gestation**									
Red blood cells 10^6^/μL	**3.6**	488	2.7	4.6	**3.8**	367	2.7	4.9	**< .0001**
Hemoglobin g/dL	**10.3**	486	7.1	12.7	**11.0**	367	8.2	13.2	**< .0001**
Hematocrit %	**30.2**	488	21.6	37.4	**32.3**	367	23.8	39.4	**< .0001**
Platelets 10^3^/μL	**227.8**	488	103	399	**204.3**	367	97	350	**< .0001**
White blood cells 10^3^/μL	**6.2**	488	2.8	9.5	**6.9**	366	3.3	11.1	**< .0001**
Lymphocytes %	**32.2**	481	19.0	62.5	**31.5**	358	19.0	57.0	**0.3**
Monocytes %	**7.7**	481	1.7	17.7	**6.8**	358	1.9	17.0	**0.004**
Granulocytes %	**59.9**	481	21.4	75.5	**61.7**	358	28.6	77.5	**0.03**
CD4+ cells/μL	**418**	489	352	495	**810**	246	344	1366	**< .0001**
CD8+ cells/μL	**788**	489	349	1602	**514**	246	262	1002	**< .0001**
CD4 %	**28.6**	49	17	52	**47.6**	55	33	65	**< .0001**
CD8 %	**51.7**	49	22	77	**31.7**	55	18	50	**< .0001**
Total T-Lymphocytes as % of all lymphocytes	**77.0**	40	46	96.5	**75.6**	47	60	93	**0.5**

**Study visit at 36 weeks gestation**									

Red blood cells 10^6^/μL	**3.8**	356	2.8	4.9	**3.9**	280	2.9	5.3	**0.002**
Hemoglobin g/dL	**10.7**	356	8	13.5	**11.3**	280	9	14.0	**< .0001**
Hematocrit %	**31.7**	356	23.3	40	**33.3**	280	25.9	41.9	**< .0001**
Platelets 10^3^/μL	**214.0**	356	86	448	**182.9**	280	87	345	**< .0001**
White blood cells 10^3^/μL	**6.1**	356	3.3	9.9	**6.7**	280	3.8	11.2	**< .0001**
Lymphocytes %	**31.6**	356	19.3	53.4	**31.8**	274	18.1	53.6	**0.8**
Monocytes %	**8.6**	356	2.5	23.5	**7.5**	274	1.1	23.1	**0.01**
Granulocytes %	**59.6**	356	29.5	75.0	**60.7**	274	32.6	76.5	**0.2**
CD4+ cells/μL	**418**	359	353	495	**786**	166	368	1325	**< .0001**
CD8+ cells/μL	**779**	359	340	1515	**507**	166	216	1106	**< .0001**
CD4 %	**28.2**	36	12	50	**45.0**	49	28	66	**< .0001**
CD8 %	**51.8**	36	20	76	**31.1**	49	21	43	**< .0001**
Total T-Lymphocytes as % of all lymphocytes	**78.4**	34	57	92	**74.1**	43	54	90	**0.05**

Laboratory values for pregnant women at the 20–24-week gestational age visit and at the 36-week visit. Of the 2633 women with data at baseline, 727 (27.6%) were missing week 36 data and 1906 (72.4%) had 36-week data. It is possible that week 36 women were slightly healthier than week 20–24 women, since the 36 week women had a slightly higher CD4+ cell count and may have been more likely to be able to keep the 36-week appointment (see discussion).

Lower 95% confidence interval = P2.75 = 2.75%ile

Upper 95% confidence interval = P97.5 = 97.5%ile

**Table 7:** Comparison of HIV-infected women with CD4+ cell counts >500/μL to HIV-uninfected women

	**HIV (+) CD4 > 500/μL**				**HIV (-)**				
**Laboratory measures**	**Mean**	N	P2.75	P97.5	**Mean**	N	P2.75	P97.5	**P value**
**Study visit at 20–24 weeks gestation**									
Red blood cells 10^6^/μL	**3.7**	512	2.8	4.8	**3.8**	367	2.7	4.9	**0.01**
Hemoglobin g/dL	**10.5**	512	7.7	13	**11.0**	367	8.2	13.2	**< .0001**
Hematocrit %	**30.9**	512	21.5	38.5	**32.3**	367	23.8	39.4	**< .0001**
Platelets 10^3^/μL	**242.9**	512	111	453	**204.3**	367	97	350	**< .0001**
White blood cells 10^3^/μL	**7.2**	513	3.5	12.0	**6.9**	366	3.3	11.1	**0.02**
Lymphocytes %	**33.7**	506	20.1	60.1	**31.5**	358	19.0	57.0	**0.0005**
Monocytes %	**7.7**	506	1.2	21.2	**6.8**	358	1.9	17.0	**0.008**
Granulocytes %	**58.2**	506	21.5	73.4	**61.7**	358	28.6	77.5	**< .0001**
CD4+ cells/μL	**670**	515	504	1090	**810**	246	344	1366	**< .0001**
CD8+ cells/μL	**893**	515	364	1919	**514**	246	262	1002	**< .0001**
CD4 %	**34.4**	56	16	51	**47.6**	55	33	65	**< .0001**
CD8 %	**48.1**	56	28	71	**31.7**	55	18	50	**< .0001**
Total T-Lymphocytes as % of all lymphocytes	**77.4**	43	55	94	**75.6**	47	60	93	**0.4**

**Study visit at 36 weeks gestation**									

Red blood cells 10^6^/μL	**3.9**	422	2.9	5.0	**3.9**	280	2.9	5.3	**0.3**
Hemoglobin g/dl	**11.0**	422	8.4	13.2	**11.3**	280	9.0	14.0	**0.003**
Hematocrit %	**32.7**	422	23.6	40.0	**33.3**	280	25.9	41.9	**0.06**
Platelets 10^3^/μL	**212.2**	422	104	382	**183**	280	87	345	**< .0001**
White blood cells 10^3^/μL	**7.1**	421	4.3	11.0	**6.7**	280	3.8	11.2	**0.01**
Lymphocytes %	**33.3**	417	18.5	50.3	**31.8**	274	18.1	53.6	**0.01**
Monocytes %	**7.9**	417	1.9	19.1	**7.5**	274	1.1	23.1	**0.4**
Granulocytes %	**58.7**	417	33.8	74.2	**60.7**	274	32.6	76.5	**0.02**
CD4+ cells/μL	**677**	425	507	1155	**786**	166	368	1325	**< .0001**
CD8+ cells/μL	**870**	425	395	1639	**507**	166	216	1106	**< .0001**
CD4 %	**35.2**	33	16	53	**45.0**	49	28	66	**< .0001**
CD8 %	**47.1**	33	29	68	**31.1**	49	21	43	**< .0001**
Total T-Lymphocytes as % of all lymphocytes	**79.3**	28	60	91	**74.1**	43	54	90	**0.02**

Laboratory values for pregnant women at the 20–24-week gestational age visit and at the 36-week visit. Of the 2633 women with data at baseline, 727 (27.6%) were missing week 36 data and 1906 (72.4%) had 36-week data. It is possible that week 36 women were slightly healthier than week 20–24 women, since the 36 week women had a slightly higher CD4+ cell count and may have been more likely to be able to keep the 36-week appointment (see discussion).

Lower 95% confidence interval = P2.75 = 2.75%ile

Upper 95% confidence interval = P97.5 = 97.5%ile

Tables 8 and 9 give the paired sample t-tests for the laboratory values between the two time points in pregnancy, among HIV-infected women and HIV-uninfected women, respectively. In both groups, the RBC count, hemoglobin, hematocrit and differential monocyte count increased between the 20–24-week and the 36-week visits.

**Table 8:** Comparison of laboratory values at 20–24 weeks vs. 36 weeks gestation among HIV-infected women

**Laboratory Parameter**	**Number of Pairs**	Mean (*) of the paired differences	**95% CI**		p value*
			**Lower**	**Upper**	
Red blood cell count 10^6^/μL	1626		0.17	0.23	< 0.0001
Hemoglobin g/dl	1623	0.5	0.4	0.6	< 0.0001
Hematocrit %	1626	1.5	1.3	1.7	< 0.0001
Platelets 10^3^/μL	1625	-23.9	-27.5	-20.4	< 0.0001
White blood cell count 10^3^/μL	1623	-0.1	-0.2	0.1	0.3
Lymphocytes %	1570	0.5	0.0	1.0	0.1
Monocytes %	1570	0.5	0.2	0.9	0.002
Granulocytes %	1569	-0.8	-1.6	-0.1	0.02
CD4+ T lymphocyte count cells/μL	1343	14.8	7.1	22.5	0.0002
CD8+ T lymphocyte count cells/μL	1343	8.5	-6.6	23.5	0.3
CD4+ T lymphocyte %	107	-2.3	-3.7	-0.8	0.003
CD8+ T lymphocyte %	107	-2.5	-4.6	-0.4	0.02
Total T lymphocytes as a % of all lymphocytes	74	-1.3	-3.7	1.1	0.3

* For each pair, the 20–24 week laboratory value is subtracted from the 36 week value. Hence, a negative value indicates a decrease over time in the laboratory parameter.

**Table 9:** Comparison of laboratory values at 20–24 weeks vs. 36 weeks gestation among HIV-uninfected women

**Laboratory Parameter**	**Number of Pairs**	Mean (*) of the paired differences	**95% CI**		p value*
			**Lower**	**Upper**	
Red blood cells count 10^6^/μL	280	0.2	0.1	0.3	< 0.0001
Hemoglobin g/dl	280	0.2	0.1	0.4	0.01
Hematocrit %	280	0.7	0.1	1.3	0.01
Platelets 10^3^/μL	280	-17.6	-25.1	-10.0	< 0.0001
White blood cell count 10^3^/μL	279	-0.2	-0.4	0.1	0.2
Lymphocytes %	266	0.1	-1.1	1.2	0.9
Monocytes %	266	0.9	0.1	1.7	0.02
Granulocytes %	266	-0.9	-2.5	0.6	0.2
CD4+ T lymphocyte count cells/μL	134	-24.2	-71.9	23.6	0.3
CD8+ T lymphocyte count cells/μL	134	-12.5	-52.5	27.5	0.5
CD4+ T lymphocyte %	25	-2.5	-6.2	1.3	0.2
CD8+ T lymphocyte %	25	-1.2	-3.6	1.3	0.3
Total T lymphocytes as a % of all lymphocytes	19	1.1	-3.1	5.2	0.6

(*) For each pair, the 20–24 week laboratory value is subtracted from the 36 week value. Hence, a negative value indicates a decrease over time in the laboratory parameter.

### Laboratory Measures in Infants

We compared hematological measures of HIV-infected versus HIV-uninfected infants at birth (Table 10) and at 4–6 weeks of age (Table 11). At birth, there were no significant differences in hemoglobin, hematocrit and white blood cell (WBC) count between HIV-infected and HIV-uninfected infants. At birth, HIV-infected infants had lower platelet counts than HIV-uninfected infants. At the 4–6-week visit, HIV-infected infants had lower hemoglobin and hematocrit values, lower platelet counts, and higher ALT values than HIV-uninfected infants. Table 12 provides the difference between the laboratory values for HIV-uninfected and HIV-infected infants at birth versus 4–6 weeks of age. Among the infants, hemoglobin, hematocrit and WBC counts decreased in the first 4–6 weeks of life, though platelet counts increased.

**Table 10:** Laboratory values among HIV-infected and HIV-uninfected infants (both exposed and unexposed to HIV) at birth

**Laboratory Parameter**	**HIV-uninfected Infants**				**HIV-infected Infants**				**P value**
	**n**	**Mean ± Standard Deviation**	**95% CI**		**n**	**Mean ± Standard Deviation**	**95% CI**		
			**Lower**	**Upper**			**Lower**	**Upper**	
Hemoglobin g/dl	1900	**18.5 ± 4.3**	10.1	28.2	149	**18.0 ± 3.7**	10.4	24.6	0.1
Hematocrit %	1897	**54.8 ± 12.1**	29.7	80.2	149	**53.5 ± 10.8**	29.8	72.3	0.2
Platelets 10^3^/μL	1893	**240.8 ± 102.7**	76	458	147	**207.0 ± 97.3**	37	419	< 0.0001
White blood cell count 10^3^/μL	1865	**15.5 ± 6.2**	5.8	30.6	145	**15.4 ± 10.5**	3.4	39.7	0.8

**Table 11:** Laboratory values among HIV-infected and HIV-uninfected infants at 4–6 weeks of age

**Laboratory Parameter**	**HIV-uninfected Infants**				**HIV-infected Infants**				**P value**
	**n**	**Mean ± Standard Deviation**	**95% CI**		**n**	**Mean ± Standard Deviation**	**95% CI**		
			**Lower**	**Upper**			**Lower**	**Upper**	
Hemoglobin g/dl	1710	**11.6 ± 2.6**	7.7	18	269	**11.2 ± 2.8**	6.6	18.6	< 0.007
Hematocrit %	1710	**34.0 ± 7.3**	22.4	51.5	269	**32.9 ± 8.5**	18	55.2	< 0.02
Platelets 10^3^/μL	1704	**371.8 ± 156.3**	90	697	269	**306.5 ± 150.7**	55	626	< 0.0001
White blood cell Count 10^3^/μL	1694	**10.7 ± 4.2**	5	20.9	268	**11.6 ± 5.3**	4.5	25.2	< 0.002
*ALT (SGPT) IU/L	1552	**22.2 ± 24.1**	5	62	272	**30.8 ± 33.9**	5	133	< 0.001

*ALT (SGPT) = alanine aminotransferase (serum glutamic pyruvic transaminase)

**Table 12:** Comparison of laboratory values at birth vs. 4–6 weeks of age among HIV-infected and HIV-uninfected infants

**Laboratory Parameter**	**HIV-infected Infants**				**HIV-uninfected Infants**				**p value**
	**Number of Pairs**	Mean (*) of the paired differences	**95% CI**		**Number of Pairs**	Mean (*) of the paired differences	**95% CI**		
			**Lower**	**Upper**			**Lower**	**Upper**	
Hemoglobin g/dl	124	-7.1	-7.8	-6.4	1301	-7.0	-7.2	-6.8	< 0.0001
Hematocrit %	124	-21.0	-23.0	-19.0	1298	-20.9	-21.6	-20.3	< 0.0001
Platelets 10^3^/μL	122	89.2	64.0	114.5	1292	125.1	115.9	134.3	< 0.0001
White blood cell count 10^3^/μL	119	-2.0	-3.4	0.5	1263	-4.8	-5.2	-4.5	0.009

(*) For each pair, the birth laboratory value is subtracted from the 4–6 week value. Hence a negative value indicates a decrease over time in the laboratory parameter.

We categorized HIV-uninfected infants as to whether they were born to HIV-infected mothers (termed HIV-exposed infants) or to HIV-uninfected mothers (termed HIV-unexposed infants). HIV-exposed, but uninfected infants had lower hemoglobin and hematocrit levels than HIV-unexposed infants both at birth (both p < 0.0001) and at 4–6 weeks (both p < 0.05; Table 13). Platelet counts did not differ, while at the visit at 4–6 weeks of infant age, both white blood cells and ALT were higher among exposed infants (Table 13).

**Table 13:** Laboratory values for all infants who were HIV-uninfected at birth (negative by PCR at 6 weeks of age), by HIV exposure status at the first birth visit.

**Laboratory measures**	**HIV-unexposed infant**				**HIV-exposed infant**				**p-value**
	**N**	**Mean**	**95% CI**		**N**	**Mean**	**95% CI**		
			**Lower**	**Upper**			**Lower**	**Upper**	
**Values at first research assessment after birth**									
Hemoglobin g/dL	275	**19.4**	10.6	28.8	1625	**18.3**	9.9	27.9	< .0001
Hematocrit %	275	**58.0**	33.0	85.8	1622	**54.2**	29.4	79.4	< .0001
Platelets 10^3^/μL	274	**247.5**	78.0	450.0	1619	**239.7**	75.0	458.0	0.2
White blood cells 10^3^/μL	272	**15.9**	6.5	33.0	1593	**15.5**	5.7	30.5	0.3
**Values at research assessment at age 4–6 weeks of age**									
Hemoglobin g/dL	278	**11.9**	8.1	18.9	1432	**11.6**	7.6	17.9	0.02
Hematocrit %	278	**34.8**	22.7	53.4	1432	**33.8**	22.3	51	0.04
Platelets 10^3^/μL	277	**383.4**	104	704	1427	**369.5**	89	697	0.2
White blood cells 10^3^/μL	276	**10.1**	5.1	17.4	1418	**10.9**	5.0	21.3	0.006
*ALT (SGPT) IU/liter	123	**16.4**	5	38	1429	**22.7**	5	63	0.005

* ALT (SGPT) = alanine aminotransferase (serum glutamic pyruvic transaminase)

HIV-exposed means that these infants were born to HIV-infected mothers, while HIV-unexposed infants were born to HIV-uninfected mothers. Alanine aminotransferase (serum glutamic pyruvic transaminase) was not performed at the birth visit.

Among HIV-uninfected infants (by PCR at 6 weeks of age), HIV-exposed infants had a 37-gram lower mean birth weight compared to HIV-unexposed infants (2980 ± 505 grams; 95% ci: 2957–3005 vs. 3079 ± 515 grams; 95% ci: 3023–3135, respectively; p = 0.002).

## Discussion and Conclusion

A four-site clinical trial in Malawi, Tanzania, and Zambia enabled us to document hematological, biochemical, and immunological measures in a large cohort of pregnant African women with and without HIV infection, and in their infants. The infants were either HIV-infected, HIV-exposed but uninfected, or were uninfected and unexposed to HIV. HIV-infected pregnant women had lower red blood cell counts, hemoglobins, hematocrits and WBC counts than the HIV-uninfected cohort. However, platelet and monocyte counts were higher in HIV-infected women than in the HIV-uninfected women. Although values were similar at birth, HIV-infected infants had lower hemoglobin, hematocrit, and platelet counts at 4–6 weeks of age, but had higher ALT values, compared to HIV-uninfected infants. Among the HIV-uninfected infants, the HIV-exposed babies had lower hemoglobins/hematocrits, but had higher white blood cell counts and ALT levels than did the HIV-unexposed infants at 4–6 weeks of age.

As one might expect, HIV-infected women were more likely to be anemic (hemoglobin <12 g/dL) than HIV-uninfected women.[1] One possible cause of anemia in these women could be the effect of HIV infection on hematopoiesis. The mean maternal hemoglobin levels in both HIV-infected and HIV-uninfected women in this study were lower than the values reported elsewhere for African pregnant women (mean = 12.1 g/dL).[1] The levels of hemoglobin in this study are similar to those reported previously for pregnant Indian women (mean = 11.1 ± 1.6 g/dL).[15] Lower hemoglobin levels in this study may be seen in individuals with malarial infection, but no recruited women had suspected malaria and all were recruited at urban sites with low prevalence rates of malaria. A Nigerian study found that anemia was twice as common in HIV-infected subjects who had malaria parasites, compared to non-parasitized controls.[16] In this study, routine malaria blood smears were not done at the time of laboratory testing. No women were on zidovudine as potent antiretroviral therapy and zidovudine for PMTCT were not in use at the time of the study. Women were supplemented with iron during pregnancy, so the true magnitude of anemia may be greater than what we report from this clinical trial.

Mean WBC counts were within the ranges of those previously described.[1] The WBC count for normal male and female adults is 4,500–11,000/μL (range is estimate of 95% confidence limits).[17] One study reported that although changes in leukocyte counts during pregnancy in African women were similar to those reported in Caucasian women, the total WBC counts were lower in the African women.[5]

Although hematological parameters were comparable at birth between HIV-infected infants and HIV-uninfected infants, HIV-infected infants showed a greater decrease in hemoglobin concentrations than HIV-uninfected infants in the first 4–6 weeks of life. Similar findings have been found in a large cohort of HIV-infected and uninfected infants in Malawi.[8] In this study, hemoglobin levels were lower among HIV-infected infants whose mothers had received NVP prophylaxis, and higher among HIV-uninfected infants who were not exposed to NVP prophylaxis. We were not able to assess whether the lower hemoglobin levels seen in HIV-infected infants in HPTN024 were due to HIV infection or NVP exposure, since nearly all HIV-infected women and their infants in this cohort received NVP prophylaxis.

HIV-infected infants had higher ALT levels than HIV-uninfected infants at 4–6 weeks of age. Increased ALT levels in infants may suggest acute liver inflammation, though ALT values are variable in infants and may be linked to NVP exposure.[8] Given near universal NVP prophylaxis in our cohort, we were not able to determine whether the higher ALT levels were related to HIV infection status per se, or NVP exposure. The higher ALT level in the HIV-exposed infants who were not infected, compared to HIV-uninfected and unexposed infants, may also have been due to NVP exposure, but this will require further study. The magnitude of the ALT rise was not substantial and the highest ALT in an HIV-exposed (and NVP-exposed) infant was only 63 IU/liter (Table 9).

The mean hemoglobin value for normal infants has been reported as 18.5 g/dL in a neonate ages 1–3 days in standard hematological references (Table 14),[17] similar to our study and a study in Malawi.[8] Perhaps the iron-containing vitamin and mineral supplements in our mothers improved infants' hemoglobin levels, as noted in a Tanzanian study.[11] These values are 2 g/dL higher than those reported in two other African studies done in Malawi and Nigeria that suggested that the hemoglobin values at birth were lower in African than Caucasian children.[2,6] The hemoglobin levels in this study were also higher than those found in a study of Italian neonates in which the mean hemoglobin level in term, appropriate-for-age neonates was only 14.4 (± 4.4) g/dL.[18] Two studies in India and one in Turkey also reported lower hemoglobin levels than found in studying our African newborns.[15,19,20] Newborn hemoglobin and hematocrit levels may be influenced by many factors, including the mode of delivery.[21] For example, if a midwife holds a baby a bit lower than the level of the mother's pelvis and the not-yet-delivered placenta immediately after birth and before the cord is clamped, the baby's hemoglobin can be raised. WBC counts in infants in HPTN024 are within the normal ranges of standard references for largely Caucasian infants whose mean WBC value for normal infants at birth is 18.1/μL (Table 14).[17]

**Table 14:** Normal blood values in neonates, reference value data largely derived from North American Caucasians.

**Red Blood Cell Values in Neonates: Mean and Lower Limit of Normal (-2 SD)**								
	**Hemoglobin (g/dL)**		**Hematocrit (%)**		**Red Cell Count (10^6^/μL)**			
Age	**Mean**	-2 SD	**Mean**	-2 SD	**Mean**	-2 SD		
Birth (cord blood)	**16.5**	13.5	**51**	42	**4.7**	3.9		
1 to 3 days (capillary)	**18.5**	14.5	**56**	45	**5.3**	4.0		
1 week	**17.5**	13.5	**54**	42	**5.1**	3.9		
1 month	**14.0**	10.0	**43**	31	**4.2**	3.0		
2 months	**11.5**	9.0	**35**	28	**3.8**	2.7		
**Mean and Range of Leukocyte Count and Differential Count Values in Neonates**								
**Neonatal age in hours (hr)**	**Total Leukocytes (10^3^/μL)**		**Neutrophils (10^3^/μL)**			**Lymphocytes (10^3^/μL)**		
	**Mean**	**Range**	**Mean**	**Range**	**%**	**Mean**	**Range**	**%**
Birth	18.1	9.0 – 30.0	11.0	6.0 – 26.0	61	5.5	2.0 – 11.0	31
12 hr	22.8	13.0 – 38.0	15.5	6.0 – 28.0	68	5.5	2.0 – 11.0	24
24 hr	18.9	9.4 – 34.0	11.5	5.0 – 21.0	61	5.8	2.0 – 11.5	31
1 month	10.8	5.0 – 19.5	3.8	1.0 – 9.0	35	6.0	2.5 – 16.5	56

from Geaghan SM. Appendix B, Normal blood values: Selected health-associated values for neonatal, pediatric, and adult populations. Greer JP, Foerster J, Lukens JN., et al, eds. Wintrobe's Clinical Hematology (12th Ed.). Philadelphia: Lippincott Williams & Wilkins, 2009: 2584–6.

Hematological and biochemical measurements can also be useful for clinical monitoring of HIV-infected individuals when viral load testing and CD4+ cell count monitoring are not readily available.[22] Whether they could be useful surrogates for pregnant women or young children is not known. There are findings that will require specific study, including the higher platelet counts noted in our HIV-infected mothers, but lower platelet counts in the HIV-infected infants, for which we do not have a ready explanation.[2,9,23-26]

Red cell and white cell indices have been reported to be affected by age, sex, diet, malnutrition, co-infection, and medication intake.[1-4,27] A limitation of this study is that these factors were not fully analyzed as potential confounders or interaction factors. We also present laboratory values for women before and after receiving micronutrient supplements, but are not certain that the differences at the 36-week visit are due to this supplementation as they were received by all women and because women seen at the 36-week visit may have been healthier than those missing this visit (by virtue of the slightly higher mean CD4+ cell count noted in the subset of women who came from their 36-week visit). Because this was a multi-center study in three countries with a large sample size, one may be tempted to assume that our results are generalizable for pregnant African women and their infants receiving NVP prophylaxis. However, our studies were in four urban centers where antenatal clients received good primary care and malaria was not contributing substantially to adverse outcomes. In addition, the effects of overall improved nutritional supplementation and upgraded antenatal care may have improved birth outcomes. For example, the comparable gestational ages of HIV-infected and HIV-uninfected infants in our study might not have been expected given the preponderance of preterm birth among HIV-infected infants reported in the literature.[28] (Preterm birth and HIV infection may be associated with reduced transplacental transfer of HIV-specific acquired maternal immunity to babies who are born early.)

Availability of hematologic and biochemical data in sub-Saharan pregnant women and their children with and without HIV infection can assist in clinical program development, clinical research design and planning, training of both health care providers, and community education. Our large study of poor pregnant women who received vitamin and nutrient supplementation and their offspring in urban Africa provides new normative data that will be useful comparison values for clinicians managing similar patients with and without HIV.

## Competing interests

The authors declare that they have no competing interests.

## Authors' contributions

KM, SHV, and RLG conceptualized the manuscript, designed the analyses, and drafted the manuscript. YQC and AM participated in the design of analyses, crafted the tables from the dataset, and performed the statistical analyses. JSR and MV oversaw the parent clinical trial and provided writing and editing input to the manuscript drafts. KM, WU, and NC collected the data and helped conceptualize the analysis. All authors read and approved the final manuscript. Each author has participated sufficiently in the work to take public responsibility for appropriate portions of the content.

## Pre-publication history

The pre-publication history for this paper can be accessed here:


